# Assessing the levels of intraspecific admixture and interspecific hybridization in Iberian wild goats (*Capra pyrenaica*)

**DOI:** 10.1111/eva.13299

**Published:** 2021-09-29

**Authors:** Tainã Figueiredo Cardoso, María Gracia Luigi‐Sierra, Anna Castelló, Betlem Cabrera, Antonia Noce, Emilio Mármol‐Sánchez, Ricardo García‐González, Alberto Fernández‐Arias, José Luis Alabart, Jorge Ramón López‐Olvera, Gregorio Mentaberre, José Enrique Granados‐Torres, Jesús Cardells‐Peris, Antonio Molina, Armand Sànchez, Alex Clop, Marcel Amills

**Affiliations:** ^1^ Department of Animal Genetics Centre for Research in Agricultural Genomics (CRAG) CSIC‐IRTA‐UAB‐UB Campus de la Universitat Autònoma de Barcelona Bellaterra Spain; ^2^ Departament de Ciència Animal i dels Aliments Universitat Autònoma de Barcelona Bellaterra Spain; ^3^ Leibniz‐Institute for Farm Animal Biology (FBN) Dummerstorf Germany; ^4^ Instituto Pirenaico de Ecología (IPE‐CSIC) Jaca Spain; ^5^ Servicio de Caza y Pesca Departamento de Agricultura, Ganadería y Medio Ambiente Gobierno de Aragón Zaragoza Spain; ^6^ Unidad de Producción y Sanidad Animal Centro de Investigación y Tecnología Agroalimentaria de Aragón (CITA) Instituto Agroalimentario de Aragón ‐ IA2 (CITA‐Universidad de Zaragoza) Gobierno de Aragón Zaragoza Spain; ^7^ Wildlife Ecology & Health Group and Servei d’Ecopatologia de Fauna Salvatge (SEFaS) Departament de Medicina i Cirurgia Animals Universitat Autònoma de Barcelona Bellaterra Spain; ^8^ Wildlife Ecology & Health Group and Departament de Ciència Animal Escola Tècnica Superior d’Enginyeria Agraria (ETSEA) Universitat de Lleida (UdL) Lleida Spain; ^9^ Wildlife Ecology & Health Group and Espacio Natural Sierra Nevada Granada Spain; ^10^ SAIGAS (Servicio de Análisis, Investigación y Gestión de Animales Silvestres) and Wildlife Ecology & Health Group, Faculty of Veterinary Universidad Cardenal Herrera‐CEU, CEU Universities Valencia Spain; ^11^ Departamento de Genética Universidad de Córdoba Córdoba Spain

**Keywords:** *Capra pyrenaica*, genetic diversity, high‐density SNP arrays, Iberian ibex, introgression

## Abstract

Iberian wild goats (*Capra pyrenaica*, also known as Iberian ibex, Spanish ibex, and Spanish wild goat) underwent strong genetic bottlenecks during the 19^th^ and 20^th^ centuries due to overhunting and habitat destruction. From the 1970s to 1990s, augmentation translocations were frequently carried out to restock Iberian wild goat populations (very often with hunting purposes), but they were not systematically planned or recorded. On the other hand, recent data suggest the occurrence of hybridization events between Iberian wild goats and domestic goats (*Capra hircus*). Augmentation translocations and interspecific hybridization might have contributed to increase the diversity of Iberian wild goats. With the aim of investigating this issue, we have genotyped 118 Iberian wild goats from Tortosa‐Beceite, Sierra Nevada, Muela de Cortes, Gredos, Batuecas, and Ordesa and Monte Perdido by using the Goat SNP50 BeadChip (Illumina). The analysis of genotypic data indicated that Iberian wild goat populations are strongly differentiated and display low diversity. Only three Iberian wild goats out from 118 show genomic signatures of mixed ancestry, a result consistent with a scenario in which past augmentation translocations have had a limited impact on the diversity of Iberian wild goats. Besides, we have detected eight Iberian wild goats from Tortosa‐Beceite with signs of domestic goat introgression. Although rare, hybridization with domestic goats could become a potential threat to the genetic integrity of Iberian wild goats; hence, measures should be taken to avoid the presence of uncontrolled herds of domestic or feral goats in mountainous areas inhabited by this iconic wild ungulate.

## 1 INTRODUCTION

The genetic diversity of wild animal species has been modified by multiple factors related with human activity. Habitat destruction and fragmentation combined with overhunting, climate change, and the introduction of invasive animals and plants have caused severe reductions in the genetic diversity and fitness of wild species, leading, in some cases, to their extinction (Fahrig, 2003; Pimm et al., 2014). Human activities, either intentionally or not, might have also contributed to increase the genetic diversity of wild species. Translocation, which implies the deliberate release of animals from one location to another with the goal of reinforcing, introducing, or reintroducing a species within its indigenous range (Griffith et al., 1989), can be effective in enhancing genetic diversity (Chipman et al., 2008). Obviously, it can also have adverse effects on resident animals at release sites, including the spread of diseases that could cause drastic population bottlenecks (Chipman et al., 2008). Moreover, increased stress and mortality of released animals may limit the potential benefits of translocations (Chipman et al., 2008).

Hybridization between wild animals and livestock herds, which is largely unintentional, can also increase the genetic diversity of wild species, by introducing completely new alleles and genotypes, at the expense of decreasing adaptive potential due to outbreeding depression and behavioral changes, for example, reduced predator and human avoidance (Barbato et al., 2017; Goedbloed et al., 2013). Even in the cases in which interspecific hybridization is a rare event, it can lead to long‐lasting changes in the genomic architecture of the affected wild species (Schwenk et al., 2008). While reduction of genetic variation mediated by humans has been documented in wild animals and its consequences have been thoroughly assessed (Abascal et al., 2016; Grossen et al., 2020), few reports have addressed the potential impact of translocation and interspecific hybridization on the genetic diversity of wild species (Shackleton, 1997).

The Iberian wild goat (*Capra pyrenaica*, also known as Iberian ibex, Spanish ibex, and Spanish wild goat) is a wild goat ungulate native to the Iberian Peninsula which inhabits mountainous and rocky areas and feeds on shrubs, bushes, and grasses (Acevedo & Cassinello, 2009a; Granados et al., 2001, 2007). According to Cabrera (1911, 1914), in the early 20^th^ century, there were four Iberian wild goat subspecies, namely *C*. *p*. *hispanica* (CPH, south and east of the Iberian Peninsula), *C*. *p*. *victoriae* (CPV, center and northwest of the Iberian Peninsula), *C*. *p*. *lusitanica* (CPL, Galicia and north of Portugal, extinct in the 19^th^ century), and *C*. *p*. *pyrenaica* (CPP, Pyrenees), which became extinct two decades ago (García‐González et al., 2021). The Iberian wild goat was abundant during the Middle Ages but it experienced a sustained and strong demographic decline during the 19–20^th^ centuries as a consequence of the growing hunting pressure (particularly during the 1940s–1970s) and habitat loss and fragmentation (García‐González, 2011; Pérez et al., 2002). The strong reduction of genetic diversity produced by this process of demographic contraction has been previously reported (Amills et al., 2004; Angelone et al., 2018). Strong signs of genetic differentiation among Iberian wild goat populations due to reproductive isolation and substantial genetic drift associated with severe genetic bottlenecks have also been described (Amills et al., 2004; Angelone et al., 2018). In the last decades, the creation of a network of national parks and protected areas, the absence of predators, reforestation policies, and the progressive abandonment of rural activities have contributed to the recovery and subsequent expansion of Iberian wild goats (Acevedo & Cassinello, 2009b).

Iberian wild goats constitute a valuable model to explore the impact of translocation and hybridization on genetic diversity. Restocking/repopulation translocations have favored gene flow between distant populations (Acevedo & Cassinello, 2009a; Crampe, 1991). The most comprehensive report to date analyzing the variability of 333 Iberian wild goats with a panel of 30 microsatellites did not show any evidence of genetic signatures typically associated with translocations and population admixture (Angelone‐Alasaad et al., 2017). However, this outcome might be caused by the limited resolution of the microsatellite panel employed in such study.

In addition, the impact of domestic goat introgression on the genetic diversity of Iberian wild goats is not well known yet. Alasaad et al. (2012) reported the mating of one captive Iberian wild goat male with domestic goats and the obtaining of viable offspring. Hybrids between Alpine ibexes (*Capra ibex*) and domestic goats have been also described (Giacometti et al., 2004). Moreover, Angelone et al. (2018) reported the segregation, in Iberian wild goats from four Southern Spain locations (Sierras de Cazorla, Segura and las Villas Natural Park, El Hosquillo in Serranía de Cuenca Natural Park, Sierra del Mencal, and Cabañeros National Park), of one major histocompatibility complex class II *DRB1* allele, MHC DRB1*7, identical to another one reported in domestic goats. They hypothesized that this result could be due to either the maintenance of ancient polymorphisms by balancing selection or, alternatively, introgressions from domestic goats through interspecific hybridization, and they concluded that this matter should be clarified in future (Angelone et al., 2018). By using a high‐throughput single nucleotide polymorphism (SNP) genotyping approach, we expect to answer this question and find out whether domestic goat introgression has had a significant impact on the genetic diversity of Iberian wild goats.

In summary, the main goal of the current work is to investigate the impact of intraspecific (translocations) and interspecific (hybridization between wild and domestic goats) gene flow on the diversity of Iberian wild goats by genotyping 118 individuals with a SNP assay.

## 2 MATERIALS AND METHODS

### 2.1 Study areas and historical description of populations

**FIGURE 1:**
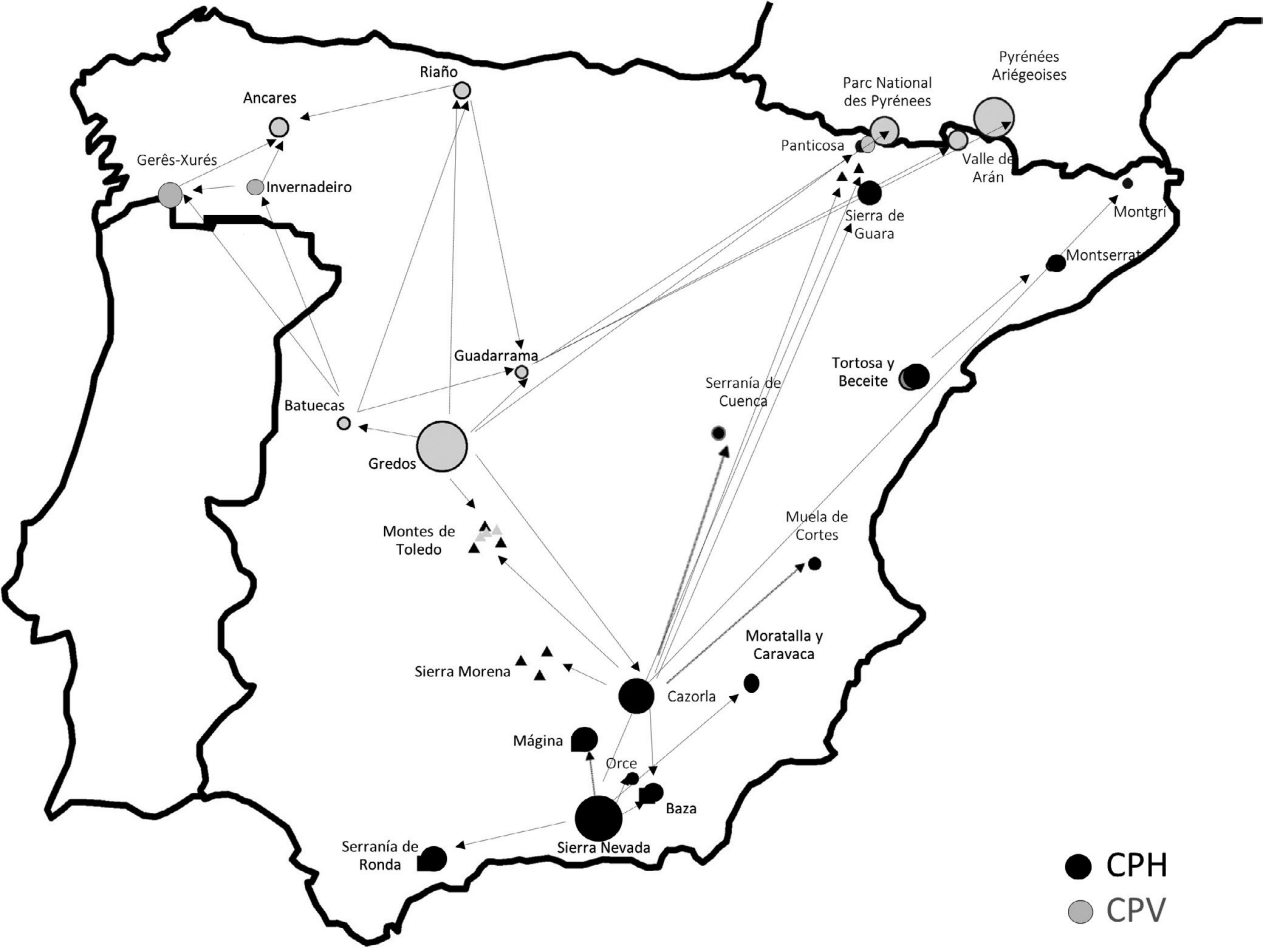
Documented translocations of Iberian wild goats: This description of Iberian wild goat translocations should be considered as partial because many historical translocations went unrecorded. With regard to *Capra pyrenaica victoriae* (CPV), 12 specimens from Gredos (Ávila) were translocated to Panticosa (Huesca, 1960s). A few years later, only 2 males and 4 females were left. While one of the males was transferred to Gredos and the other one died, the whereabouts of the females are unknown although it is highly unlikely that they reached Ordesa (García‐González, 1989). Iberian wild goats from Gredos were also translocated to Las Batuecas (Salamanca, 1970s), Regional Hunting Reserve of Riaño (León), and La Pedriza (current Sierra de Guadarrama National Park, Madrid‐Segovia) during the 90s. Before 1995, the introduction of specimens from Gredos to private farms in the Montes de Toledo is also documented (Acevedo et al., 2011). There were also translocations of individuals from Batuecas to Sierra de Guadarrama (Madrid) and to Riaño (León). The Natural Park of Invernadero also received Iberian wild goats from Batuecas (Prada & Herrero, 2013) and possibly from Gredos (Crampe, 2020), although this latter translocation event is not completely confirmed. An unsuccessful reintroduction attempt was made, between 1957 and 1962, in the Covadonga National Park (Asturias) with 14 individuals from Gredos and Cazorla (Jaen), as reported by Arenzana et al. (1964). At the end of the 90s, translocations from Riaño to Los Ancares (León) and, in 2005–2007, to Mampodre (León) are also known. In 2018, CPV individuals from Guadarrama were transferred to the Pyrenees National Park and the Ariège Pyrenees Regional Park, both in France, and to the Valle de Arán (Lleida). Concerning *Capra pyrenaica hispanica* (CPH), Iberian wild goats from Cazorla were taken to private farms in the Montes de Toledo, Sierra Morena (Jaén‐Ciudad Real), Serranía de Cuenca (Hosquillo), Sierra de Guara (Huesca), Sierra de Baza (Granada), and Muela de Cortes (Valencia) during the 1960s and 1970s. More recently animals from Cazorla were transferred to an enclosure in the Serra del Montgrí (Girona), but they escaped and formed a population of more than one hundred specimens. At the end of the nineties, Iberian wild goats were taken from Tortosa to Montserrat (Barcelona). Finally, CPH from Sierra Nevada were introduced in the Serranía de Ronda (Málaga) and in the Sierra de Baza (Granada) during the 1970s and 1980s, and to Sierra de Mágina (Jaén). CPH from Sierra Nevada were also brought to enclosures in Almuñecar (Granada), Garcipollera (Huesca), and Cumbres Mayores (Huelva) at the end of the 90s. Finally, during the first decade of this century, Iberian wild goats from the Sierra Nevada have been brought to the Moratalla and Caravaca mountains in the Murcia region and Sierra de Orce in the north of the province of Granada

In this work, we have investigated three CPH populations (Tortosa‐Beceite, Muela de Cortes, and Sierra Nevada), two CPV populations (Gredos and Batuecas) and one CPP individual (Ordesa and Monte Perdido). All these Iberian wild goat populations underwent strong bottlenecks during the 19^th^ and 20^th^ centuries but, as shown in Table 1, during the last six decades they have experienced an accelerated demographic expansion due to the lack of predators, human depopulation in rural areas, and protected status (Acevedo & Cassinello, 2009b). Hybridization with domestic goats has not been reported in any of the five populations mentioned before. While the cohabitation of domestic goats and Iberian wild goats has been described as a risk factor for the transmission of certain diseases (Astorga Márquez et al., 2014), to the best of our knowledge the spatial proximity between wild and domestic goat populations has not been thoroughly investigated in Spain. Part of the translocations among Iberian wild goat populations have been documented, and such information can be found in Figure 1.

**TABLE 1:** Current and past sizes of Iberian wild goat population investigated in the current work

Ssp^a^	Population	Past size	Current size	References
CPH	Tortosa‐Beceite	450 (1966)	~4000 (2008)	Casanovas‐Urgell et al. (2008) and Angelone et al. (2018)
	Sierra Nevada	450 (1960)	15,000 (2020)	Angelone et al. (2018)
	Muela de Cortes	?	1400 (2014)	Tinoco‐Torres et al. (2014)
CPV	Gredos	10 (1905)	~13,000 (2018)	Angelone et al. (2018)
	Batuecas	200 (1980)	900 (2002)	Pérez et al. (2002)
CPP	Ordesa and Monte Perdido	6–14 (1990)	Extinct (2000)	García‐González and Herrero (1999)

^a^ Iberian wild goat subspecies: CPH = *C*. *p*. *hispanica*; CPV = *C*. *p*. *victoriae*; CPP = *C*. *p*. *pyrenaica*.

### 2.2 Isolation of genomic DNA from Iberian wild goat samples

We used two different batches of CPV and CPH samples. The first batch was reported by Jiménez et al. (1999), as well as by Amills et al. (2004), and consisted of (1) CPV: liver samples from seven and 14 Iberian wild goats from Batuecas and Gredos, respectively; (2) CPH: blood samples from 27 and five Iberian wild goats from Tortosa‐Beceite and Sierra Nevada, respectively, and five liver samples from Iberian wild goats inhabiting Muela de Cortes. A second batch included blood or solid tissue (muscle, spleen, or ear cartilage) samples from 59 CPH individuals from Tortosa‐Beceite (*N* = 43, 2010–2019), Muela de Cortes (*N* = 7, 2017–2019), and Sierra Nevada (*N* = 9, 2006–2014). Finally, one muscle sample from one of the last CPP representatives was collected in the location of Ordesa and Monte Perdido in 1996, before the extinction of this subspecies. Genomic DNA was isolated from blood samples as previously reported (Amills et al., 1996), while a standard phenol–chloroform protocol was used to purify genomic DNA from solid tissues (Sambrook & Russell, 2006). The second batch of samples and the CPP sample had not been analyzed in previous genetic studies.

### 2.3 Genotyping with the Goat SNP50 BeadChip (Illumina)

Following the instructions of the manufacturer, we genotyped with the Goat SNP50 BeadChip from Illumina (Tosser‐Klopp et al., 2014) samples from 118 Iberian wild goats. The Goat SNP50 BeadChip (Illumina) includes 53,348 SNPs with an approximately uniform distribution in the caprine genome (Tosser‐Klopp et al., 2014). The GenomeStudio software (Illumina) was employed to call genotypes and to assess sample and genotype qualities by using a cluster file provided by the International Goat Genome Consortium (cluster file: GoatIGGC_Cons_60k.egt, Tosser‐Klopp et al., 2014). In GenomeStudio, genotypes can be visualized as Genoplots. For each sample, genotypes are called by their signal intensity (norm R) and allele frequency (Norm Theta) relative to canonical cluster positions for the SNP marker under study (https://www.illumina.com/techniques/microarrays/array‐data‐analysis‐experimental‐design/genomestudio.html).

Preprocessing and filtering of data were carried out with the PLINK v.1.7 software (Purcell et al., 2007). More specifically, markers with a GenTrain score (Illumina descriptive statistic related to clustering quality) lower than 0.8, unmapped SNPs, as well as SNPs that mapped to the X chromosome on the goat reference genome assembly *Capra hircus*—ARS1 (Bickhart et al., 2017, https://www.ensembl.org/Capra_hircus/) and those with a minimum allele frequency (MAF) lower than 0.01 (‐maf 0.01) were filtered out. Markers with an individual missingness rate with more than 50% missing genotypes SNPs (–mind 0.5) and SNP with a missingness across samples greater than 1% (–geno 0.01) were also removed. After applying these filtering criteria, 21,621 SNPs were retained for genetic analyses.

To compare the diversity of Iberian wild goats and domestic goats, we used a previously published caprine data set corresponding to 50 domestic goats (ten individuals per breed) from Northern Spain (Bermeya and Blanca de Rasquera) and Southern Spain (Florida, Malagueña, and Murciano‐Granadina) typed with the Goat SNP50 BeadChip (Illumina) by Manunza et al. (2016).

### 2.4 Population genetics analyses

The majority of the 21,621 SNPs which passed the filtering criteria only segregated in eight Iberian wild goats from Tortosa‐Beceite, probably because of their introgression by domestic goats (see Section 3). Given that most Iberian wild goats were monomorphic for this set of SNPs (Figure S1), we generated a second set of SNPs by excluding the eight individuals referred above and considering the same filtering criteria defined in the previous section. After this, a set of 1001 SNPs was obtained. Population genetics analyses were based on both sets of SNPs depending on their goals. The analyses targeting specifically the eight putative hybrid individuals were carried out with the set of 21,621 SNPs, while analyses comprising the eight hybrid and the 110 non‐hybrid Iberian wild goats were based on the set of 1001 SNPs (because the remaining 20,620 SNPs are monomorphic in the non‐hybrid individuals so they cannot be used). For the sake of clarity, the dataset used for each one of the analysis carried out in our study is specified below. We considered as putative hybrids the eight individuals that did not collapse in the MDS plot shown in Figure S1 and that, in addition, showed signatures of domestic goat introgression in the admixture analyses (see below). The remaining 110 Iberian wild goats were considered as non‐hybrids, although we cannot completely rule out the possibility that a number of them may carry a domestic goat genetic component not detectable with our methods.

### 2.5 Multidimensional scaling and estimation of diversity parameters in Iberian wild goats

The PLINK v1.7 software (Purcell et al., 2007) was used to carry out sample clustering based on the multidimensional scaling (MDS) of allele information from retrieved SNPs (–cluster –mds‐plot 2 eigendecomp eigvals). We did four MDS analyses: (1) only Iberian wild goat populations (1001 SNPs, *N* = 118); (2) only Iberian wild goat populations (*N* = 118) with a data set of 894 SNPs, *that is*, 1001 SNPs minus the SNPs with missing values in the CPP sample (the individual with the highest genotype missingness rate). The reason for doing this is that MDS analyses tend to "locate" samples close to the center when missingness is high, so we wanted to test whether this circumstance could affect our results; (3) Iberian wild goat and domestic goat populations (1001 SNPs, *N* = 118 Iberian wild goats, *N* = 50 domestic goats); (4) eight hybrid Iberian wild goats from Tortosa‐Beceite and 50 domestic goats (21,621 SNPs). MDS plots were built in R software by using the ggplot2 package (Wickham, 2016).

Observed (*H_o_*) and expected (*H_e_*) heterozygosities, as well as the inbreeding coefficient *F*_hat2_, were calculated by using the PLINK v.1.7 software (Purcell et al., 2007) and the data set of 1001 SNPs. We chose *F*_hat2_ as an estimate of inbreeding because, in a previous study focused on domestic goats, this statistic showed a high correlation (*r* = 0.88, *p*‐value = 1.00E‐04) with F_ROH_ (Cardoso et al., 2018). In contrast with other inbreeding coefficients, *F*_hat2_ can take negative values (when the count of observed homozygotes is lower than the expected count of homozygotes) because it is not defined as a probability but as an excess of homozygosity‐based inbreeding estimate (Purcell et al., 2007). The –hardy command was used to compute *H_o_* and *H_e_*, while the –ibc command was used to estimate the *F*_hat2_ coefficient. Nucleotide diversity was computed for each population on a per‐site basis (π, command: –site‐pi) using the VCFtools software (Danecek et al., 2011). Confidence intervals (CI) for each parameter were calculated according to the following formula:

(1) $$\text{CI =}\;\bar{X}\pm 1\text{.96*SE}$$

where $\bar{X}$ is the sample mean of the parameter for each population, 1.96 is the *Z*‐score corresponding to a 95% confidence interval, and SE is the standard error of the mean (Sim & Reid, 1999).

Genome‐wide identity by descent (IBD) between pairs of samples was estimated with the PI‐HAT coefficient, which describes the probability of sharing 0, 1, or 2 alleles IBD by pairs of individuals from the same homogeneous random‐mating population (Purcell et al., 2007). Heatmap plots were built in R software by using the ggplot2 package (Wickham, 2016).

### 2.6 Examining the ancestry of Iberian wild goats with admixture

We used the Admixture software (Alexander et al., 2009) to calculate maximum likelihood estimates of individual ancestries from SNP data generated with the Goat SNP50 BeadChip (Illumina) considering Iberian wild goat and domestic goat populations (1001 SNPs, *N* = 118 Iberian wild goats, *N* = 50 domestic goats). A cross‐validation fold at 5% and a block bootstrap with 2000 iterations were used to calculate SE for admixture proportions. Confidence intervals for admixture proportions were inferred with Equation 1. The optimal K‐value was the one with the lowest cross‐validation error, as determined with the method of Alexander and Lange (2011). The Pophelper package for R (Francis, 2017) was used to process the output results from the Admixture analysis.

### 2.7 Performance of an f3 test of admixture in eight putative hybrid Iberian wild goats

In the Admixture analysis, eight individuals from Tortosa‐Beceite showed genomic signatures of introgression by domestic goats. We used the qp3pop program, included in the ADMIXTOOLS software package (Patterson et al., 2012), and the set of 21,621 SNPs to carry out a 3‐population test in the form f3(admixed Tortosa‐Beceite; Tortosa‐Beceite, Malagueña), that is, we selected the non‐admixed Tortosa‐Beceite and Malagueña individuals as representatives of Iberian wild goats and domestic goats, respectively. In the absence of hybridization, f3 has a non‐negative mean, while a negative mean is expected in the case of hybridization. The statistical significance of the result can be assessed by means of a *Z*‐score. In order to evaluate the robustness of the f3 results *vs* the choice of particular source populations, we did two additional analyses in the form f3(admixed Tortosa‐Beceite; Gredos, Bermeya) and f3(admixed Tortosa‐Beceite; Batuecas, Malagueña).

## 3 RESULTS

### 3.1 About the polymorphism of domestic goat SNP markers in Iberian wild goats

**FIGURE 2:**
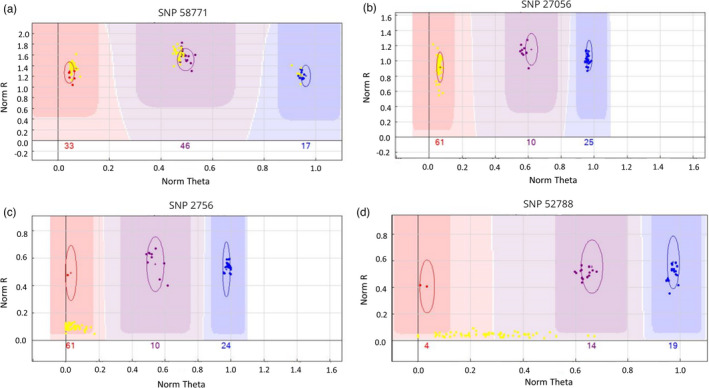
Four representative Genoplots obtained with the GenomeStudio software through the analysis of Goat SNP50 BeadChip (Illumina) data corresponding to Iberian wild goats (yellow) and domestic goats (red, purple, and blue). Genotypes are called for each sample (dots) by taking into account their signal intensity (Norm R, *y*‐axis) and allelic intensity ratio (Norm Theta, *x*‐axis) relative to canonical cluster positions (dark shading). (a) Genoplot showing the genotypes obtained for SNP 58771. It can be seen that this SNP is successfully called and it segregates in both the Iberian wild goat and domestic goat populations. (b) Genoplot showing the genotypes obtained for SNP 27056. This SNP is successfully called and it segregates in domestic goats (two genotypes are called) but not in Iberian wild goats (a single genotype is called). The intensity of the signal (norm R) is similar in domestic goats and Iberian wild goats. (c) Genoplot showing the genotypes obtained for SNP 2756. This SNP is successfully called and it segregates in domestic goats (three genotypes are called) but not in Iberian wild goats (a single genotype is called and with a weaker intensity than the one corresponding to domestic goats). (d) Genoplot showing the genotypes obtained for SNP 52788. This SNP is not successfully called in Iberian wild goats (norm R is much lower than that observed in domestic goats)

The call rates of Iberian wild goats genotyped with the Goat SNP50 BeadChip (Illumina) ranged between 0.94 and 0.99, with an average of 0.98 ± 0.04. The only exception was the CPP DNA extracted from a muscle sample, which was considerably degraded and displayed a call rate of 0.86. In Figure 2, we show four representative examples of the Genoplots generated with the GenomeStudio software. The analysis of the Goat SNP50 BeadChip (Illumina) genotype data revealed the existence of 21,621 SNPs arrayed in the Goat SNP50 BeadChip (Illumina) that segregated (MAF > 0.01) in the 118 Iberian wild goats under investigation. This high proportion (~40%) of SNPs shared by domestic goats and Iberian wild goats is consistent with the introgression of Iberian wild goats by domestic goats. The MDS obtained with the 21,621 SNPs revealed that all Iberian wild goats, with the exception of eight individuals from Tortosa‐Beceite, collapsed in a single location of the plot (Figure S1). This outcome is produced by the fact that the majority of Iberian wild goats are monomorphic for most of the Goat SNP50 BeadChip (Illumina) markers. The second SNP filtering procedure excluding the eight putative hybrid individuals reported above yielded a drastic reduction of the number of domestic goat SNPs segregating (MAF > 0.01) in Iberian wild goats, that is, from 21,621 to 1001 markers. Thus, these 1001 SNP markers were polymorphic (MAF > 0.01) in the non‐hybrid Iberian wild goats as well as in the eight hybrids individuals from Tortosa‐Beceite. In contrast, 20,620 markers were polymorphic only in the eight putative hybrid individuals.

### 3.2 Population structure and diversity of Iberian wild goats

**FIGURE 3:**
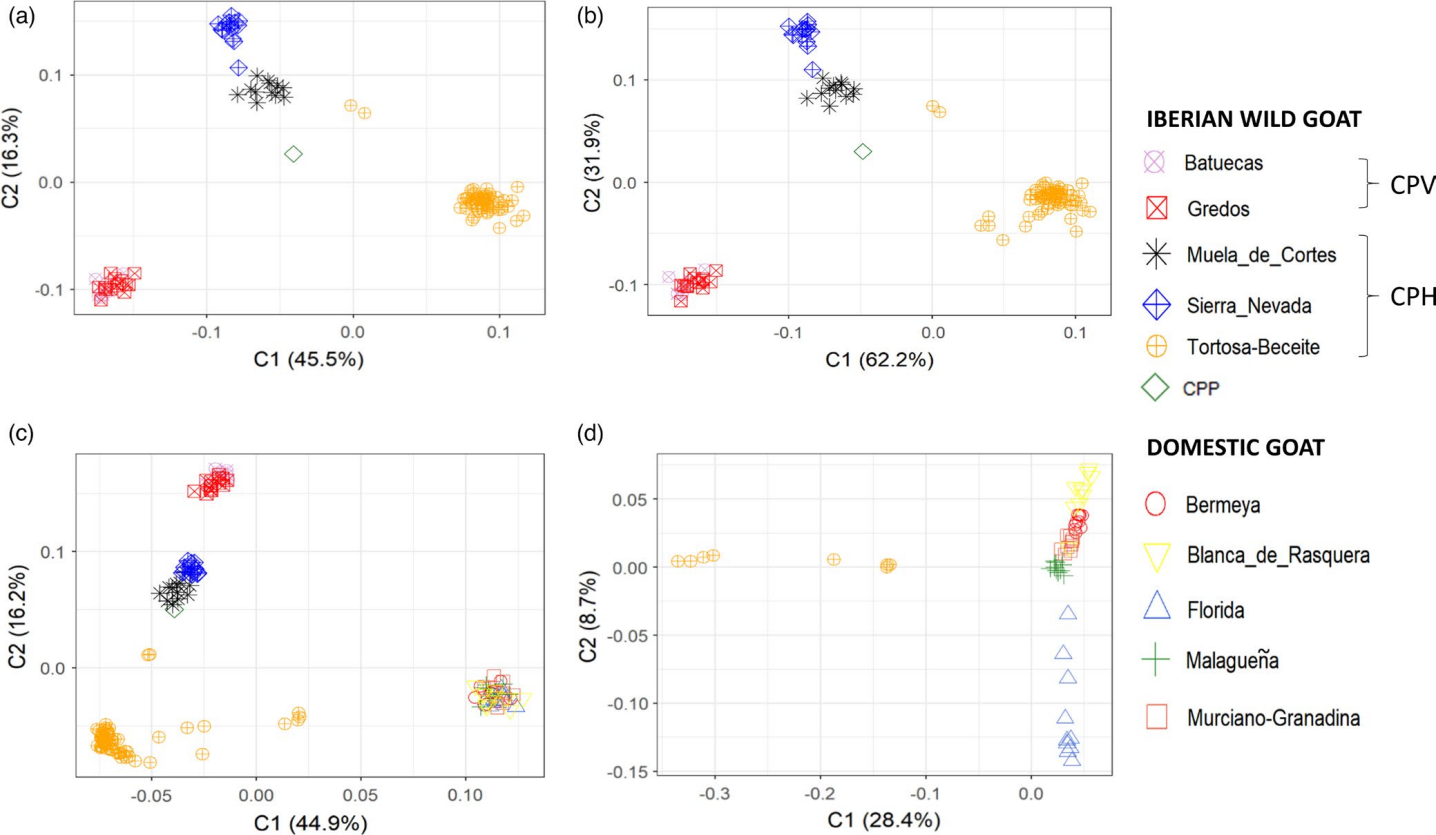
Multidimensional scaling (MDS) plot of Iberian wild goats. (a) MDS plot including 118 Iberian wild goat samples. This analysis is based on 1001 SNPs from the Goat SNP50 BeadChip (Illumina), (b) MDS of Iberian wild goat individuals. This MDS plot includes 118 Iberian wild goat samples and it is based on 894 SNPs from the Goat SNP50 BeadChip (Illumina), that is, 1001 SNPs minus the SNPs showing missing values in the CPP sample (the individual with the highest genotype missingness rate). We carried out this analysis because we wanted to test whether the centrality of the CPP sample in the MDS is caused by its high missingness rate (MDS analyses tend to "locate" samples close to the center when missingness is high). By comparing a and b, it becomes clear that this is not the case. (c) MDS plot including Iberian wild goat and domestic goat populations (1001 SNPs, *N* = 118 Iberian wild goats, *N* = 50 domestic goats), and (d) MDS plot including the eight hybrid Iberian wild goats from Tortosa‐Beceite and domestic goat populations (*N* = 50). This analysis is based on 21,621 SNPs from the Goat SNP50 BeadChip (Illumina). Iberian wild goat individuals were sampled in Batuecas (*N* = 7, *Capra pyrenaica victoriae*), Gredos (*N* = 14, *Capra pyrenaica victoriae*), Tortosa‐Beceite (*N* = 70, *Capra pyrenaica hispanica*), Muela de Cortes (*N* = 12, *Capra pyrenaica hispanica*), and Sierra Nevada (*N* = 14, *Capra pyrenaica hispanica*) and National Park of Ordesa and Monte Perdido (CPP, *Capra pyrenaica pyrenaica*, *N* = 1). Goats belonged to the Bermeya, Blanca de Rasquera, Florida, Malagueña and Murciano‐Granadina Spanish breeds (*N* = 10 for each breed)

We have investigated the population structure of 118 Iberian wild goats with 1001 markers segregating in both non‐hybrid and putative hybrid individuals. We did not use the remaining 20,620 markers because this would have caused a very strong bias in our diversity estimates since all of them were monomorphic in the majority (*N* = 110) of Iberian wild goats. As expected, the MDS plot based on the information provided by these 1001 SNPs showed no signs of tight aggregation of Iberian wild goats in a single location (Figure 3a). Instead, we observed the existence of three main clusters comprising samples from (1) Tortosa‐Beceite, (2) Sierra Nevada and Muela de Cortes, and (3) Gredos and Batuecas, which showed a close correspondence with the geographic distribution of these populations (Figure 1). We also observed one individual from Sierra Nevada and two individuals from Tortosa‐Beceite located close to the Muela de Cortes cluster. The only representative of the CPP extinct population was also placed near to the Muela de Cortes cluster. We made a second analysis excluding 107 SNPs that were missing in the CPP sample (Figure 3b) to make sure that the relatively high missingness rate of the CPP sample was not affecting its position in the MDS. This analysis, based on 894 SNPs, was completely consistent with the one shown in Figure 3a. We made a third MDS analysis, also based on 1001 SNPs, and comprising both Iberian wild goat and domestic goat populations (Figure 3c). This analysis evidenced eight Iberian wild goats from Tortosa‐Beceite which were separated from the Tortosa‐Beceite cluster. These individuals correspond to the eight putative Iberian wild goat × domestic goat hybrids which did not collapse in the MDS depicted in Figure S1 (see previous section). Finally, we made a fourth analysis focused on the eight individuals mentioned above and 50 domestic goats (Figure 3d) which revealed that these eight individuals are relatively close to the Malagueña goats, although such result should be taken with caution.

As expected, the genetic diversity of Iberian wild goats was lower than that of domestic goats (Table 2). The observed (*H_o_*) and expected (*H_e_*) heterozygosities were not substantially different in the five sampled Iberian wild goat populations (Table 2). The Batuecas and Sierra Nevada populations showed the lowest *H_o_* and *H_e_* values while Muela de Cortes and Tortosa‐Beceite had the highest ones. The exclusion of the eight putative hybrid individuals from the Tortosa‐Beceite population caused ~15% reductions in *H_o_* and *H_e_* despite the fact that they just represent 6.8% of the individuals sampled in this population (Table 2). Iberian wild goats showed lower nucleotide diversity (π = 0.181) than domestic goats (π = 0.402). Consistent with heterozygosity measurements, Sierra Nevada and Batuecas populations had the lowest nucleotide diversity values (π ≅ 0.160) among the investigated populations.

**TABLE 2:** Means of diversity parameters (and their 95% confidence intervals) estimated in five Iberian wild goat populations and five domestic goat breeds on the basis of 1001 SNPs typed with the Goat SNP50 BeadChip (Illumina)^a^

Species	Population	*N*	*H_o_*	*H_e_*	π	*F*_hat2_
Iberian wild goat (*Capra pyrenaica*)	Tortosa‐Beceite (without 8 hybrid individuals)	62	0.167 (0.156–0.178)	0.174 (0.162–0.186)	0.176 (0.174–0.177)	0.593 (0.588–0.598)
	Tortosa‐Beceite (with 8 hybrid individuals)	70	0.194 (0.183–0.204)	0.198 (0.187–0.209)	0.199 (0.198–0.201)	0.516 (0.501–0.0.530)
	Muela de Cortes	12	0.202 (0.187–0.216)	0.192 (0.180–0.205)	0.211 (0.209–0.213)	0.442 (0.440–0.445)
	Sierra Nevada	14	0.153 (0.140–0.166)	0.157 (0.145–0.169)	0.160 (0.150–0.180)	0.590 (0.570–0.610)
	Gredos	14	0.181 (0.168–0.195)	0.169 (0.157–0.182)	0.176 (0.174–0.177)	0.489 (0.487–0.491)
	Batuecas	7	0.165 (0.151–0.179)	0.153 (0.141–0.165)	0.165 (0.163–0.166)	0.539 (0.537–0.541)
	Total	117	0.177	0.174	0.181	0.528
Domestic goat (*Capra hircus*)	Bermeya	10	0.404 (0.392–0.415)	0.385 (0.378–0.393)	0.406 (0.404–0.406)	−0.184 (−0.186 to −0.182)
	Blanca de Rasquera	10	0.391 (0.380–0.402)	0.377 (0.369–0.385)	0.397 (0.396–0.398)	−0.139 (−0.144 to −0.133)
	Florida	10	0.408 (0.396–0.420)	0.373 (0.365–0.382)	0.393 (0.392–0.394)	−0.188 (−0.190 to −0.186)
	Malagueña	10	0.412 (0.401–0.424)	0.395 (0.387–0.402)	0.414 (0.414–0.416)	−0.205 (−0.207 to −0.203)
	Murciano‐Granadina	10	0.395 (0.384–0.407)	0.382 (0.374–0.3990)	0.402 (0.401–0.403)	−0.164 (−0.167 to −0.161)
	Total/mean	50	0.402	0.382	0.402	−0.176

^a^ The single CPP sample has not been included in this analysis. *N* = number of individuals, *H_o_* = observed heterozygosity, *H_e_* = expected heterozygosity, π = nucleotide diversity estimated on a per‐site basis, *F*_hat2_ = excess homozygosity‐based inbreeding coefficient (it takes negative values when the observed homozygote count is lower than the expected homozygote count).

As shown in Table 2, the inbreeding *F*_hat2_ coefficient calculated with PLINK v.1.7 (Purcell et al., 2007) reached high values in the Sierra Nevada (*F*_hat2_ = 0.590) and Batuecas (*F*_hat2_ = 0.539) populations, while in the Muela de Cortes population the magnitude of the *F*_hat2_ coefficient was 20%–28% lower (*F*_hat2_ = 0.442). The values of the *F*_hat2_ coefficient in the Tortosa‐Beceite population were notably different depending on whether the eight hybrid individuals were included (*F*_hat2_ = 0.516) or not (*F*_hat2_ = 0.593) in the calculation (Table 2). In contrast, inbreeding *F*_hat2_ coefficients values were close to zero in all five domestic goat breeds (Table 2). It could be argued that 1001 SNPs are not enough to obtain reliable estimates of diversity parameters. To check this issue, we estimated *H_o_*, *H_e_*, π, and *F*_hat2_ in our data set of 50 domestic goats by using a set of 53,325 SNPs. By inspecting Table S1, it can be seen that diversity parameters estimated with either 53,325 SNPs or 1001 SNPs display fairly consistent values, with the only exception of *F*_hat2_ coefficients, which display negative (Table 2) or close to zero (Table S1) values when using sets of 1001 SNPs or 53,325 SNPs, respectively.

As shown in Table 3, genetic differentiation among Iberian wild goat populations (*F*_ST_ > 0.287) was much higher than among Spanish domestic goat breeds (*F*_ST_ < 0.072), with the only exception of the Gredos and Batuecas populations which had a weak genetic differentiation (*F*_ST_ = 0.035). The magnitude of the genetic differentiation did not correlate well with the assignment of populations to the CPH or CPV subspecies. For instance, the *F*_ST_ coefficient between the Sierra Nevada and Tortosa‐Beceite populations (*F*_ST_ = 0.379), which belong to the same CPH subspecies, was similar to *F*_ST_ coefficients measured between CPH and CPV populations. In addition, genetic differentiation among Spanish domestic goat and non‐hybrid Iberian wild goat was higher (*F*_ST_ = 0.337) than between Spanish domestic goat and the eight putative hybrid Iberian wild goats (*F*_ST_ = 0.183). The *F*_ST_ coefficient between the non‐hybrid Iberian wild goat populations and the eight Tortosa‐Beceite hybrids was 0.122.

**TABLE 3:** Estimates of *F*_ST_ coefficients between five Iberian wild goat populations^a^ and between five domestic goat breeds on the basis of 1001 SNPs typed with the Goat SNP50 BeadChip (Illumina)

Iberian wild goats					
Population	Batuecas	Gredos	Muela de Cortes	Sierra Nevada	Tortosa‐Beceite
Batuecas	0.000				
Gredos	0.035	0.000			
Muela de Cortes	0.390	0.371	0.000		
Sierra Nevada	0.465	0.445	0.287	0.000	
Tortosa‐Beceite	0.415	0.400	0.306	0.379	0.000

Domestic goats					
Population	Bermeya	Florida	Malagueña	Murciano‐Granadina	Blanca de Rasquera
Bermeya	0.000				
Florida	0.072	0.000			
Malagueña	0.047	0.0509	0.000		
Murciano‐Granadina	0.054	0.0709	0.034	0.000	
Blanca de Rasquera	0.049	0.067	0.044	0.058	0.000

^a^ The single CPP sample has not been included in this analysis.

**FIGURE 4:**
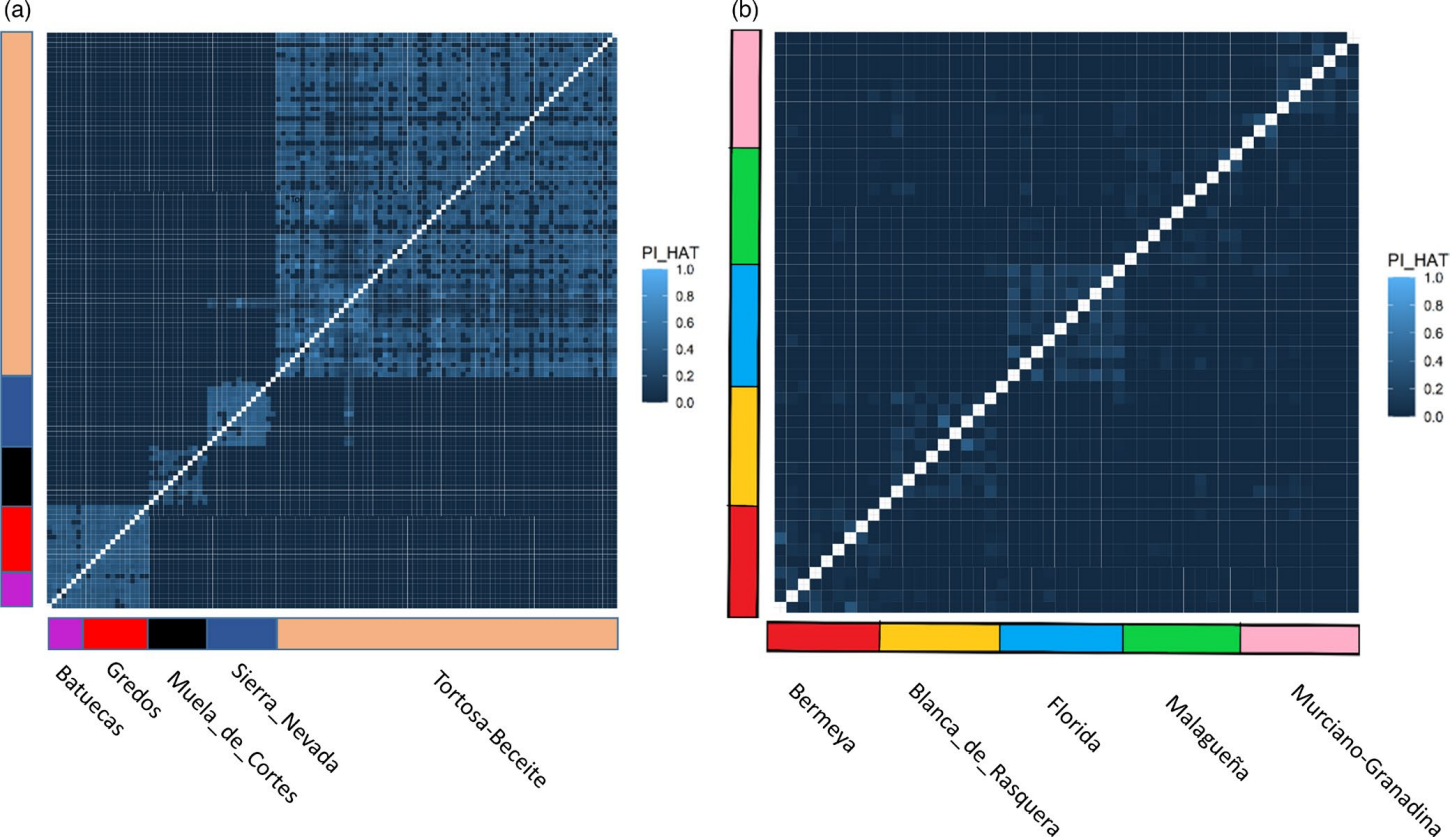
Heatmaps representing genome‐wide identity by descent (IBD) between pairs of samples as estimated with the PI‐HAT coefficient. (a) Heatmap of PI‐HAT coefficients calculated from all Iberian wild goat pairs of samples (CPP is not included in this analysis because it is represented by just 1 sample). Iberian wild goat individuals were sampled in Batuecas (*N* = 7, *Capra pyrenaica victoriae*), Gredos (*N* = 14, *Capra pyrenaica victoriae*), Tortosa‐Beceite (*N* = 70, *Capra pyrenaica hispanica*), Muela de Cortes (*N* = 12, *Capra pyrenaica hispanica*), and Sierra Nevada (*N* = 14, *Capra pyrenaica hispanica*). (b) Heatmap of PI‐HAT coefficients calculated from all domestic goat pairs of samples. Domestic goats belonged to the Bermeya, Blanca de Rasquera, Florida, Malagueña and Murciano‐Granadina breeds (*N* = 10 for each breed)

We also inferred the degree of genome‐wide IBD among Iberian wild goats (Figure 4a) and among domestic goats (Figure 4b). PI‐HAT coefficients took zero values for most pairwise comparisons among Iberian wild goats from different populations (except for the Gredos *vs* Batuecas comparison), indicating the absence of relatedness. In contrast, when these pairwise comparisons were made at the within‐population level (Figure 4a), the degree of genetic similarity among individuals increased substantially. In domestic goats, PI‐HAT coefficients reached values close to zero for the majority of pairwise comparisons, and even when they comprised individuals drawn from the same breed (Figure 4b).

### 3.3 Examining the ancestry of Iberian wild goats and detecting genomic signatures of admixture

**FIGURE 5:**
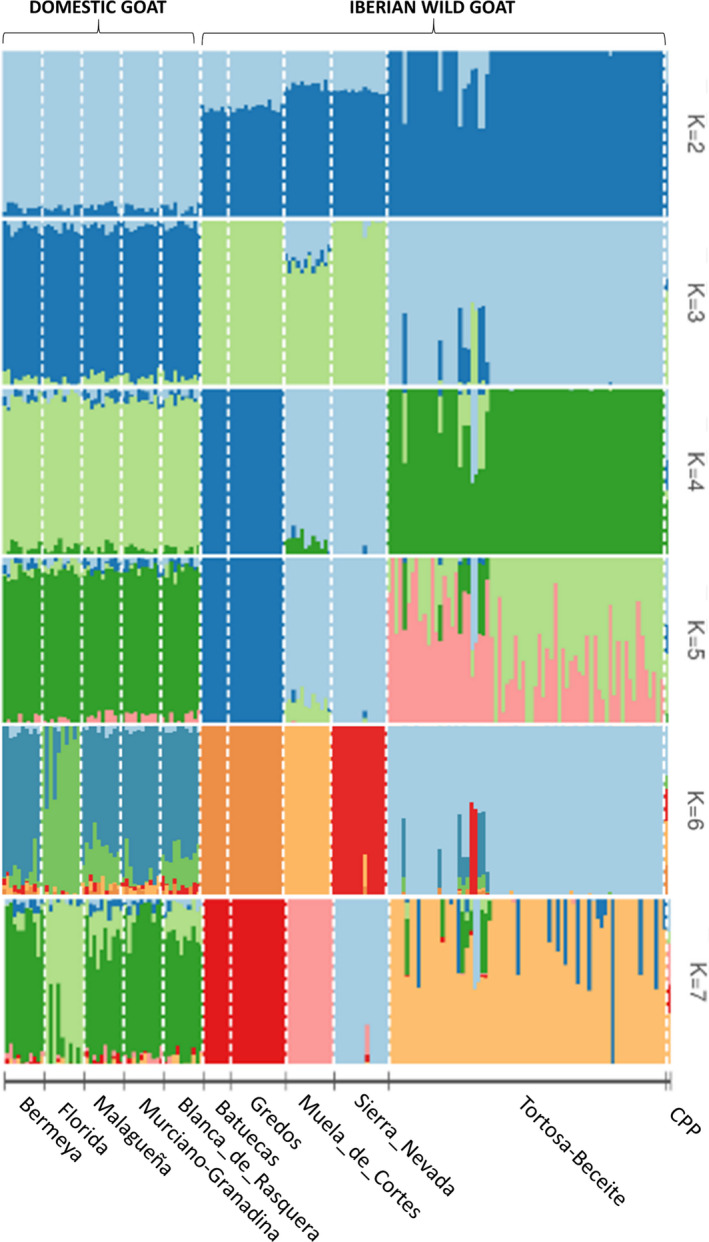
Admixture analysis of the 118 Iberian wild goats and 50 domestic goats. Each individual is represented by a single column divided into K colored segments, where K is the number of assumed clusters. Populations are separated by white lines. This analysis is based on the information provided by 1001 SNPs. The K‐ value with the lowest cross‐validation error is 7. Iberian wild goat individuals were sampled in Batuecas (*N* = 7, *Capra pyrenaica victoriae*), Gredos (*N* = 14, *Capra pyrenaica victoriae*), Tortosa‐Beceite (*N* = 70, *Capra pyrenaica hispanica*), Muela de Cortes (*N* = 12, *Capra pyrenaica hispanica*), Sierra Nevada (*N* = 14, *Capra pyrenaica hispanica*) and National Park of Ordesa and Monte Perdido (CPP, *Capra pyrenaica pyrenaica*, *N* = 1). Goats belonged to the Bermeya, Blanca de Rasquera, Florida, Malagueña and Murciano‐Granadina breeds (*N* = 10 for each breed)

The results of the Admixture analysis corresponding to 118 Iberian wild goats and 50 domestic goats characterized with 1001 SNPs are shown in Figure 5 (*K* = 2–7, *K* = 7 is the number of clusters with the lowest cross‐validation error, Figure S2). The percentages of ancestry for each one of the Iberian wild goat populations are displayed in Figure S3 for *K* = 7. In the majority of Iberian wild goat populations, admixture was low or non‐detectable (Figure 5 and Figure S3). The only exception were Iberian wild goats from Gredos and Batuecas, which showed signs of a common ancestry even at *K* = 7 (Figure 5). The CPP sample also seemed to have different ancestries (Figure 5 and Figure S3) but in this case, results are not reliable because allelic frequencies cannot be inferred from a single individual. According to the Admixture analysis, two individuals from Tortosa‐Beceite (Tortosa‐Beceite_22 and Tortosa‐Beceite_23) showed evidence of having Sierra Nevada ancestry, while one individual from Sierra Nevada (Sierra_Nevada_9) displayed signs of Muela de Cortes ancestry (Figure 5). We did not calculate f3‐statistics for these three potentially admixed Iberian wild goats because they cannot be reliably estimated with just 1001 SNPs.

### 3.4 Performance of an f3 test of admixture in eight putative hybrid Iberian wild goats

**FIGURE 6:**
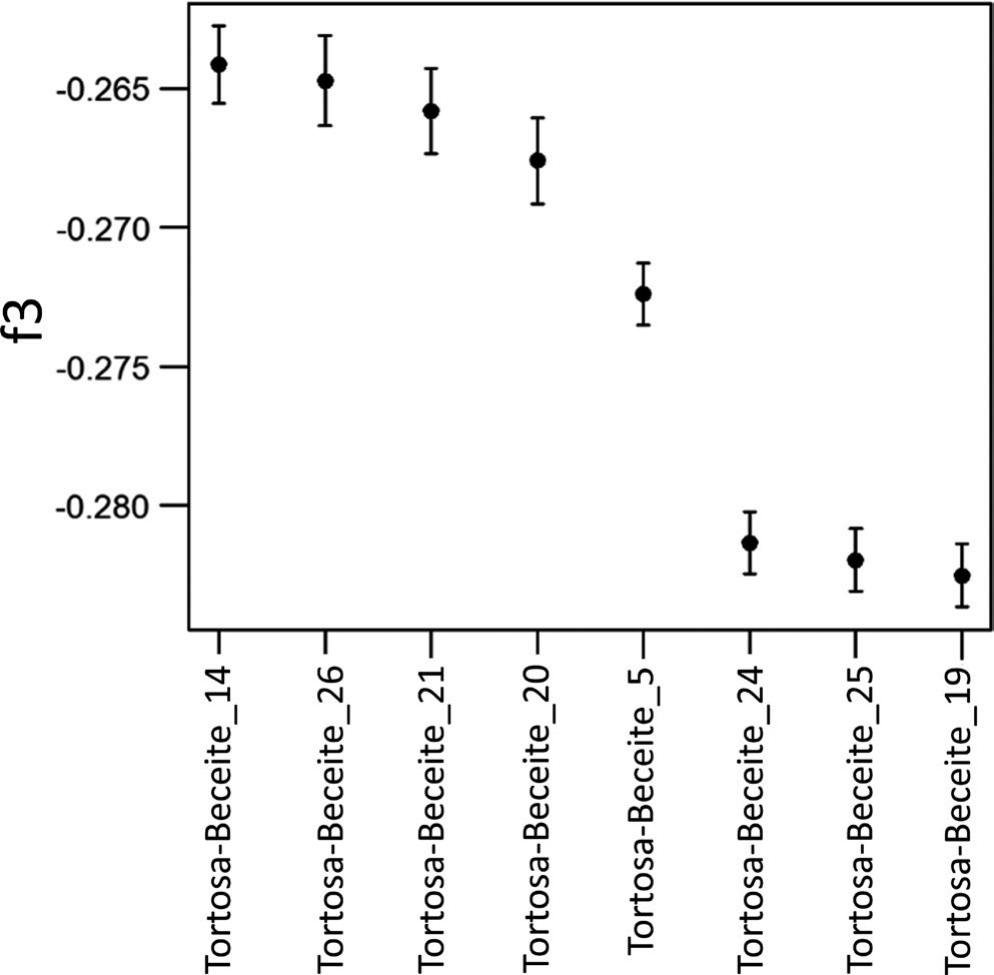
Measurement of f3‐statistics. This analysis has been carried out in the eight Iberian wild goats from Tortosa‐Beceite showing signatures of domestic goat introgression in the Admixture analysis. We computed f3‐statistics of the form f3(hybrid Tortosa‐Beceite; Tortosa‐Beceite, Malagueña) with the set of 21,621 SNPs. Error bars represent the standard errors. It can be seen that all eight f3‐statistics are negative, thus providing evidence that the eight individuals under investigation are hybrids between Iberian wild goats and domestic goats

The eight putative hybrids from Tortosa‐Beceite displayed negative f3 values, indicative of admixture between the two Tortosa‐Beceite and Malagueña source populations (Figure 6). The *Z*‐scores were high and significant (Table S2). These results were consistent even when different source populations were selected, for example, with Gredos and Bermeya, and Batuecas and Malagueña as source populations (Table S3).

## 4 DISCUSSION

### 4.1 Reduced diversity in Iberian wild goats

In the current work, we have evaluated the applicability of a high‐density SNP array, the Goat SNP50 BeadChip (Illumina), to investigate the diversity of Iberian wild goats. The call rates obtained with the Goat SNP50 BeadChip (Illumina) were consistent with previous estimates obtained by Miller et al. (2012), who reported call rates of 0.98 when using the Ovine SNP50 BeadChip (Illumina) in wild sheep species. In our study, only 1.8% of the SNPs contained in the Goat SNP50 BeadChip (Illumina) were polymorphic (MAF >0.01) in the 110 non‐hybrid Iberian wild goats. This result was expected because call rates and shared polymorphic sites decrease at linear and exponential rates, respectively (Miller et al., 2012). Based on the data presented by Miller et al. (2012), domestic goats and Iberian wild goats, two species that diverged 1.5 Mya (Lalueza‐Fox et al., 2005), should share, on average, less than 10% polymorphic sites.

In accordance with the data reported by Amills et al. (2004), we have detected reduced observed and expected heterozygosities in Iberian wild goats when compared to domestic goats (Table 2). Nucleotide diversities were also lower in Iberian wild goats than in domestic goats. Moreover, nucleotide diversities of domestic goats were similar to those observed in other livestock populations (Luigi‐Sierra et al., 2020; Wang et al., 2018). Nucleotide diversity estimated on a per‐site basis (–site‐pi), as we have done in the current work, yields values that are different from those obtained by considering windows of 1000 bp (–window‐pi), as previously discussed by Luigi‐Sierra et al. (2020). Obviously, the reduced diversity of Iberian wild goats when compared to domestic goats could be due, at least in part, to ascertainment bias. Noteworthy, Grossen et al. (2018) genotyped more than 100,000 SNPs in Alpine ibexes, Iberian wild goats, and domestic goats and found that *H_o_* and *H_e_* were twofold higher in domestic goats when compared to Alpine ibexes or Iberian wild goats, a result fully consistent with ours. Moreover, Grossen et al. (2020) reported that Iberian wild goats have twice lower genome‐wide heterozygosity (expressed as the number of heterozygous autosomal SNPs per kb) than domestic goats, a result that again matches very well ours.

Diversity across the five Iberian wild goat populations studied was similar, a result that is consistent with previous microsatellite data (Amills et al., 2004; Angelone‐Alasaad et al., 2017). These findings indicated that the bottlenecks suffered by this species during the 19^th^ and 20^th^ centuries had widespread effects on its overall genetic variation (Amills et al., 2004; Pérez et al., 2002). Moreover, *F*_hat2_ coefficients reached values of 0.442–0.593 in the Iberian wild goats, while in the domestic goats they were close to zero (Table 2), a result consistent with previous reports (Cardoso et al., 2018). The PI‐HAT values calculated in pairwise comparisons were also substantially higher in Iberian wild goats than in domestic goats, reflecting a significant proportion of IBD between pairs of Iberian wild goats coming from the same population (Figure 4a). As a reference, the PI‐HAT values estimated at the within‐population level in domestic goats (Figure 4b) were comparable to those reported in domestic sheep from Switzerland (Burren et al., 2014) as well as in domestic goats from South Africa (Visser et al., 2016).

As we have discussed previously, it could be argued that ascertainment bias might have distorted to some extent the estimation of *F*_hat2_ and PI‐HAT coefficients in Iberian wild goats. Indeed, Angelone‐Alasaad et al. (2017) reported inbreeding *F*_IS_ coefficients close to zero in Iberian wild goat populations from Maestrazgo, Sierra Nevada, and Gredos. Similarly, Bozzuto et al. (2019) described *F*_IS_ coefficients close to zero in Alpine ibex populations, but they attributed this finding to the inherent lack of power of this individual inbreeding coefficient when estimated with limited molecular data. Through a RAD‐Seq approach, Grossen et al. (2018) demonstrated that the median total length of long ROH (>5 Mb) was much higher in Iberian wild goats from Sierra Nevada (228 Mb) and Maestrazgo (179 Mb) than in domestic goats (<10 Mb). This result was further confirmed by Grossen et al. (2020), who demonstrated that the median of the proportion of the genome covered by ROH >2.5 Mb is approximately twice higher in Iberian wild goats than in domestic goats. These findings indicate that the decreased diversity and increased inbreeding of Iberian wild goats, as compared to domestic goats, observed in our study is not an artifact entirely produced by ascertainment bias. Indeed, the existence of inbreeding and low variation in Iberian wild goats is consistent with the dramatic genetic bottlenecks suffered by this ungulate species (Amills et al., 2004).

### 4.2 Iberian wild goat populations are strongly differentiated

The classification of Iberian wild goats in four subspecies proposed by Cabrera (1911, 1914) raised controversy because it relied exclusively on a limited number of highly variable phenotypic traits recorded in a low number of individuals (Angelone‐Alasaad et al., 2017). Our study demonstrated that the *F*_ST_ coefficients among Iberian wild goat populations were much higher (*F*_ST_ > 0.287) than those measured among domestic goat populations (*F*_ST_ < 0.072), and the latter were similar to those reported by Manunza et al. (2016) for the same populations using a data set of 39,257 SNPs. Moreover, genetic differentiation between the CPV and CPH populations had a magnitude similar to that observed between the Tortosa‐Beceite and Sierra Nevada CPH populations (Table 3).

Overall, these results indicated that the classification of Cabrera (1911, 1914) is not well supported by genetic evidence. Consistently, Angelone‐Alasaad et al. (2017) analyzed 333 Iberian wild goats with a panel of microsatellites and found that the CPH populations from Sierra Nevada and Maestrazgo had a degree of genetic differentiation similar to that observed among CPH and CPV specimens. The strong genetic differentiation between Iberian wild goat populations, irrespective of their assignment to one subspecies or another, is probably due to intense drift associated with past genetic bottlenecks combined with prolonged geographic isolation (Pérez et al., 2002). The only exception to this general trend were the CPV populations of Gredos and Batuecas, which had a weak degree of genetic differentiation (*F*_ST_ = 0.035, Table 3), probably because the Batuecas population originated from restockings with individuals from Gredos (Pérez et al., 2002).

In the MDS analyses shown in Figure 3a,b, the only CPP individual did not cluster with its CPV and CPH counterparts, although it was located close to the Muela de Cortes population (CPH). This result also needs to be interpreted with caution because allele frequencies cannot be estimated from just one individual. However, CPP suffered strong population bottlenecks and an irreversible genetic erosion that enhanced genetic differentiation before extinction (García‐González & Herrero, 1999). Moreover, there was no recent gene flow between CPP and the other CPV and CPH subspecies, which strengthened the progressive genetic differentiation of CPP. The analysis of additional museum CPP samples would be needed to accurately characterize the genetic relationships between this extinct subspecies and CPH and CPV.

### 4.3 Genomic signatures of past restocking translocations are rarely detected in current Iberian wild goat populations

As pointed out by Acevedo and Cassinello (2009a), Iberian wild goat distribution is the result of both natural and artificial expansion processes. Most translocations were carried out after 1970, particularly during the 1980s and 1990s (Acevedo & Cassinello, 2009a). Although there is a general consensus indicating that Iberian wild goats were the subject of numerous restocking/repopulation translocations (movement of individuals into a population of conspecifics), the majority of them are poorly documented (Angelone‐Alasaad et al., 2017). In Figure 1, we provide a description of translocations that have been reported so far.

One of the main goals of the current work was to infer whether past restocking translocation processes have left genomic signatures that can be identified in current Iberian wild goat populations, as well as to ascertain the impact of restocking/repopulation on the within‐population genetic diversity. In a previous study based on a panel of 30 microsatellites (Angelone‐Alasaad et al., 2017), no evidence of admixed individuals was obtained in any of the three Iberian wild goat populations under study. In our work, resolution and sensitivity to identify introgressed individuals were expected to be much higher because our set of 1001 SNPs would be roughly equivalent to a panel of ~300 microsatellites (Fernández et al., 2013; Herráeza et al., 2005). We have detected two individuals from Tortosa‐Beceite showing evidence of Sierra Nevada ancestry (Figure 5), and one individual from Sierra Nevada harbored a genomic signature of Muela de Cortes ancestry. Landscape fragmentation and large geographic distances between Sierra Nevada versus Muela de Cortes (300 km) and Tortosa‐Beceite (more than 500 km) make difficult to attribute this result to a natural expansion of Iberian wild goats. More probably, the presence of admixed individuals was the consequence of human‐mediated translocations. To the best of our knowledge, no translocation between these three geographic areas is documented (Figure 1), thus supporting the notion that translocation events involving Iberian wild goats went often unrecorded (Angelone‐Alasaad et al., 2017). This lack of recording might reflect that many translocations involving Iberian wild goats were not carefully planned or executed to achieve a long‐term goal. In any case, this lack of reliable data makes it difficult to infer the impact of the officially registered restocking/repopulation translocations by comparing the genetic diversity of populations before and after they were implemented. Despite this important caveat, the low proportion of individuals (~3%) with genomic signatures of mixed ancestry is consistent with a scenario in which past restocking/repopulation translocations did not have a strong impact on the genetic variability of Iberian wild goats. We do not think that this finding is produced by the limited ability of the panel of 1001 SNPs to detect admixed individuals because Iberian wild goat populations are highly differentiated, a feature that compensates to some extent the limited number of available markers, thus making it possible to detect recent admixture with enough confidence.

Many factors may explain why augmentation translocations left a scarce genomic footprint on the genomes of Iberian wild goats. For instance, chronic stress produced by the capture, handling, transport, captivity, and release of wild animals to a new location might result in substantially reduced reproductive success and increased mortality (Dickens et al., 2010). Another factor could be competition for food resources between the released animals and the residing conspecific population, or endemic diseases that might have decimated the incoming individuals. Avian translocations have a high failure rate (Dickens et al., 2009), and documented success in carnivores is also low (Macdonald, 2009). In the case of the American marten population of Wisconsin, augmentation provided minimal genetic and demographic rescue contributions (Manlick et al., 2017). A failed reintroduction of 14 Iberian wild goats from Gredos and Cazorla to the National Park of Covadonga in 1957–1962 has been also reported (Arenzana et al., 1964). Despite their low diversity, Iberian wild goat populations are increasing in numbers at a fast pace (Acevedo & Cassinello, 2009a; Acevedo et al., 2007). In light of this, we consider that gene flow between naturally expanding populations could be established without the need of human intervention.

### 4.4 Hybridization with domestic goats as a potential threat to the conservation of Iberian wild goats

We detected eight Iberian wild goats from Tortosa‐Beceite with genomic signatures of domestic goat introgression. The f3 test agreed with the results of the Admixture analysis by showing that domestic goat introgression was significant (Figure 6). The absence of hybrid individuals in Sierra Nevada, Gredos, Batuecas, or Muela de Cortes might indicate that the introgression of Iberian wild goats by domestic goats is a sporadic event. However, the sampling of several of these locations was quite limited and quantitative inferences on the abundance of hybrid individuals could not be made with high confidence.

Angelone et al. (2018) detected the segregation of a domestic goat MHC‐DRB1*7 allele in Iberian wild goats from the Sierras de Cazorla, Segura, and Las Villas (Jaén, Andalusia). They concluded that this could be due to either the maintenance of ancient polymorphisms by balancing selection or, alternatively, introgressions from domestic goats through interspecific hybridization (Angelone et al., 2018). Our results support this latter interpretation. Giacometti et al. (2004) documented the existence of free‐ranging Alpine ibexes in the Bregaglia Valley of southern Switzerland which displayed signs of domestic goat introgression. The occurrence of such interspecific hybridization events was confirmed by Grossen et al. (2014) by demonstrating that one of the two major histocompatibility complex class *DRB* alleles that segregates in Alpine ibexes was identical to another one described in domestic goats. When the National Game Reserve of Tortosa‐Beceite was created in 1966, there was an estimated population of 200 Iberian wild goats. Although domestic goats were less abundant than domestic sheep, the presence of both domestic and feral goats combined with the low number of Iberian wild goats (Viñas‐Borrell et al., 1993) originated a window of opportunity for the occurrence of interspecific hybridization events.

According to our data, the introgression of Iberian wild goats by domestic goats does not seem to be widespread, probably because the interbreeding of these two species in the wild is a rare event, and moreover, some degree of reproductive incompatibility may exist (Herrero, Fernández‐Arberas, Prada, & García‐Serrano, 2013). However, hybridization cannot be disregarded as a potential threat to the genetic conservation of Iberian wild goats, since its prevalence might increase as a result of the rapid expansion, in numbers and geographic range, of Iberian wild goat populations (Acevedo & Cassinello, 2009; Acevedo et al., 2007; Perea et al., 2015). The introgression of Iberian wild goats by domestic goats could imply a decrease of reproductive potential and fitness, the introduction of maladaptive alleles, a reduction or loss of genetic integrity, and it may also have legal implications regarding individual or population conservation status (Leonard et al., 2013). Transmission of infectious diseases by domestic goats is another factor that could have important adverse effects on the viability of Iberian wild goat populations (Brennan et al., 2014). Of particular concern are feral goats, which can adapt quite successfully to mountainous habitats (Herrero, Fernández‐Arberas, Prada, García‐Serrano, & García‐González, 2013). For instance, in the Sierra de Guara, a population of Iberian wild goats coexists with almost one thousand feral goats (Herrero, Fernández‐Arberas, Prada, García‐Serrano, & García‐González, 2013) descending from individuals probably abandoned by their owners. Extensive field surveys based on SNP markers should be conducted to evaluate the presence and frequency of hybrid individuals in current Iberian wild goat populations, with special emphasis on those inhabiting geographic areas in which the presence of uncontrolled herds of feral goats is well documented.

## CONFLICT OF INTERESTS

The authors declare that they have no competing interests.

## Supporting information

Supplementary Material

## Data Availability

The data that support the findings of this study are openly available in Figshare at https://doi.org/10.6084/m9.figshare.11955981.v2.
