# Supplementary material for: Assessing the levels of intraspecific admixture and interspecific hybridization in Iberian wild goats (Capra pyrenaica)
Source: Evol Appl. 2021 Sep 29;14(11):2618–34. doi: 10.1111/eva.13299 (PMC8591326; doi:10.1111/eva.13299)

**Suppl. Table 1.** Diversity parameters (mean and 95% confidence interval) estimated in five domestic goat breeds on the basis of 53,325 SNPs typed with the Goat SNP50 BeadChip.

| **Population** | **N** | **H_o_** | **H_e_** | **π** | ***F*_hat2_** |
| --- | --- | --- | --- | --- | --- |
| Bermeya | 10 | 0.398  (0.397 – 0.400) | 0.398  (0.396 – 0.398) | 0.398  (0.396 – 0.399) | 0.030  (0.022 – 0.038) |
| Blanca de Rasquera | 10 | 0.380  (0.378 – 0.382) | 0.378  (0.376 – 0.379) | 0.393  (0.391 – 0.393) | 0.093  (0.049 – 0.137) |
| Florida | 10 | 0.403  (0.402 – 0.404) | 0.391  (0.389 – 0.392) | 0.388  (0.386 – 0.388) | 0.021  (0.007 – 0.036) |
| Malagueña | 10 | 0.415  (0.413 – 0.416) | 0.413  (0.411 – 0.414) | 0.414  (0.413 – 0.415) | -0.020  (-0.031 – -0.009) |
| Murciano-Granadina | 10 | 0.395  (0.395 – 0.399) | 0.398  (0.398 – 0.401) | 0.399  (0.398 – 0.400) | 0.028  (0.002 – 0.053) |
| TOTAL/MEAN | 50 | 0.398 | 0.395 | 0.398 | 0.030 |

N = number of individuals, H_o_ = observed heterozygosity, H_e_ = expected heterozygosity, π = nucleotide diversity calculated on a per-site basis, F*_hat2_* = inbreeding coefficient calculated with PLINK.

**Suppl. Table 2.** Measurement of the f3-statistic in 8 Tortosa-Beceite hybrid individuals by assuming as source non-admixed Tortosa-Beceite (Iberian wild goat) and Malagueña (domestic goat) populations (all Z-scores are highly significant, *P*-value < 0.00001).

| Source  (non-admixed) | Source | Target | f3 | SE^1^ | Z |
| --- | --- | --- | --- | --- | --- |
| Tortosa_Beceite | Malagueña | Tortosa_Beceite_19 | -0.283 | 0.001 | -249.419 |
| Tortosa_Beceite | Malagueña | Tortosa_Beceite_25 | -0.282 | 0.001 | -249.175 |
| Tortosa_Beceite | Malagueña | Tortosa_Beceite_24 | -0.281 | 0.001 | -251.977 |
| Tortosa_Beceite | Malagueña | Tortosa_Beceite_5 | -0.272 | 0.001 | -245.326 |
| Tortosa_Beceite | Malagueña | Tortosa_Beceite_20 | -0.268 | 0.002 | -172.750 |
| Tortosa_Beceite | Malagueña | Tortosa_Beceite_21 | -0.266 | 0.002 | -173.880 |
| Tortosa_Beceite | Malagueña | Tortosa_Beceite_26 | -0.265 | 0.002 | -163.811 |
| Tortosa_Beceite | Malagueña | Tortosa_Beceite_14 | -0.264 | 0.001 | -191.143 |

^1^SE: standard error of the f3 estimate

**Suppl. Table 3.** Measurement of the f3-statistics in 8 Tortosa-Beceite hybrid individuals by assuming as source either Gredos and Bermeya or Batuecas and Malagueña populations (all Z-scores are highly significant, *P*-value < 0.00001).

| **Source** | **Source** | **Target** | **f3** | **SE1** | **Z** |
| --- | --- | --- | --- | --- | --- |
| Gredos | Bermeya | Tortosa-Beceite_20 | -0.287 | 0.001 | -216.827 |
| Gredos | Bermeya | Tortosa-Beceite_25 | -0.286 | 0.001 | -225.182 |
| Gredos | Bermeya | Tortosa-Beceite_26 | -0.286 | 0.001 | -218.556 |
| Gredos | Bermeya | Tortosa-Beceite_5 | -0.277 | 0.001 | -231.373 |
| Gredos | Bermeya | Tortosa-Beceite_21 | -0.268 | 0.002 | -143.488 |
| Gredos | Bermeya | Tortosa-Beceite_14 | -0.266 | 0.002 | -162.823 |
| Gredos | Bermeya | Tortosa-Beceite_19 | -0.266 | 0.002 | -152.673 |
| Gredos | Bermeya | Tortosa-Beceite_24 | -0.265 | 0.002 | -138.051 |
| Batuecas | Malagueña | Tortosa-Beceite_19 | -0.280 | 0.001 | -233.646 |
| Batuecas | Malagueña | Tortosa-Beceite_24 | -0.279 | 0.001 | -234.512 |
| Batuecas | Malagueña | Tortosa-Beceite_25 | -0.279 | 0.001 | -231.748 |
| Batuecas | Malagueña | Tortosa-Beceite_5 | -0.270 | 0.001 | -233.490 |
| Batuecas | Malagueña | Tortosa-Beceite_20 | -0.260 | 0.002 | -137.133 |
| Batuecas | Malagueña | Tortosa-Beceite_14 | -0.259 | 0.002 | -159.537 |
| Batuecas | Malagueña | Tortosa-Beceite_21 | -0.259 | 0.002 | -142.546 |
| Batuecas | Malagueña | Tortosa-Beceite_26 | -0.256 | 0.002 | -124.369 |

^1^SE: standard error of the f3 estimate

**Suppl. Fig. 1.** Multidimensional scaling plot (MDS) of Iberian wild goats. MDS plot including 118 Iberian wild goat samples from Batuecas (N = 7, *Capra pyrenaica victoriae*), Gredos (N = 14, *Capra pyrenaica victoriae*), Tortosa-Beceite (N = 70, *Capra pyrenaica hispanica*), Muela de Cortes (N = 12*, Capra pyrenaica hispanica*), Sierra Nevada (N = 14, *Capra pyrenaica hispanica*) and National Park of Ordesa y Monteperdido (CPP, *Capra pyrenaica pyrenaica*, N = 1). This analysis is based on 21,621 SNPs from the Goat SNP50 BeadChip (this is the number of domestic goat SNPs segregating in Iberian wild goats when the eight hybrid individuals are taken into account). It can be seen that the majority of Iberian wild goats cluster in a single point because they are monomorphic for most of SNPs.


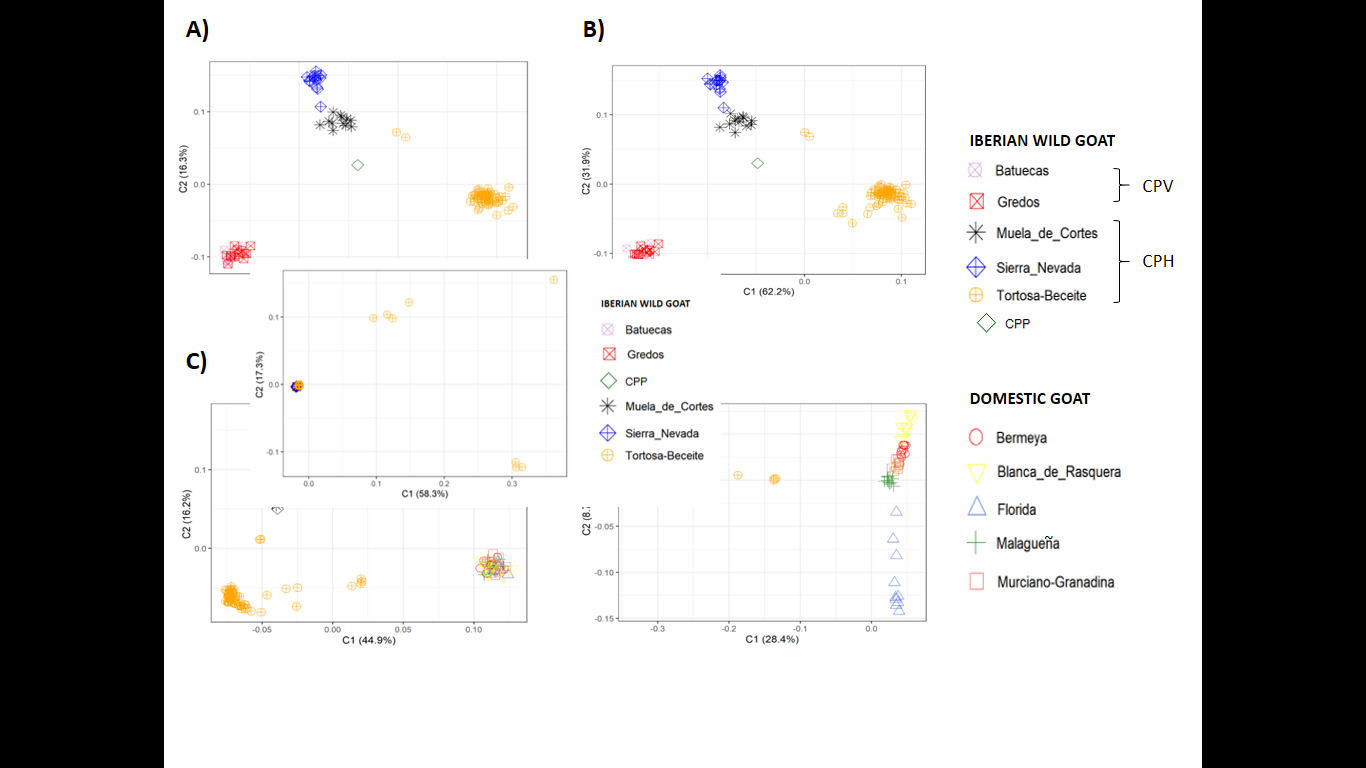


**Suppl. Fig. 2.** Plot of the cross-validation (CV) error calculated with the ADMIXTURE software for each K-value. Admixture analyses were performed with Iberian wild goat and domestic goat populations dataset (1,001 SNPs, N = 118 Iberian wild goats, N = 50 domestic goats).


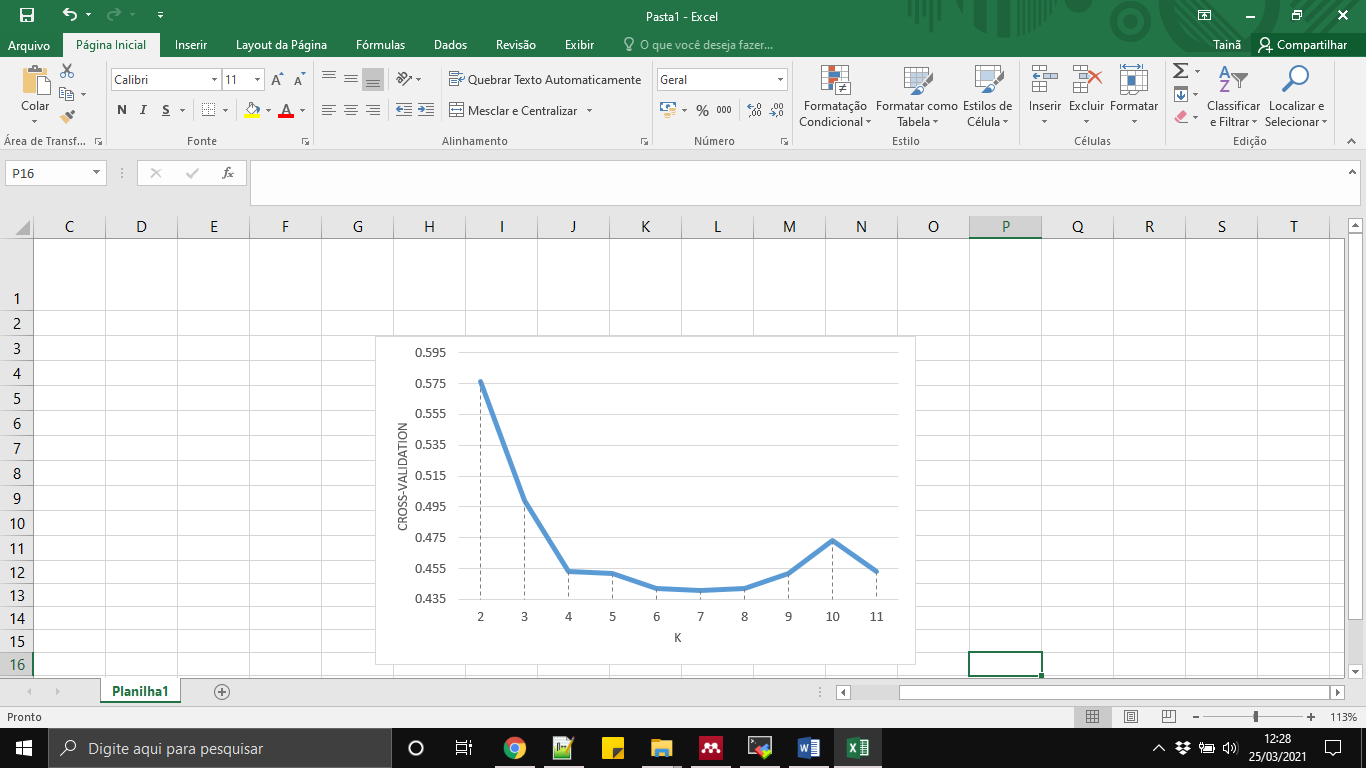


**Suppl. Fig. 3. Averaged ancestry proportions (with their 95% confidence intervals) of Iberian wild goats and domestic goats**. For this analysis, we considered the K-value with the lowest cross-validation error (K = 7). Iberian wild goat individuals were sampled in Batuecas (N = 7, *Capra pyrenaica* *victoriae*), Gredos (N = 14, *Capra pyrenaica victoriae*), Tortosa-Beceite (N = 70, *Capra pyrenaica hispanica*), Muela de Cortes (N = 12, *Capra pyrenaica hispanica*) and Sierra Nevada (N = 14, *Capra pyrenaica hispanica*) and National Park of Ordesa y Monteperdido (CPP, *Capra pyrenaica pyrenaica*, N = 1). Goats belonged to the Bermeya, Blanca de Rasquera, Florida, Malagueña and Murciano-Granadina Spanish breeds (N = 10 for each breed).


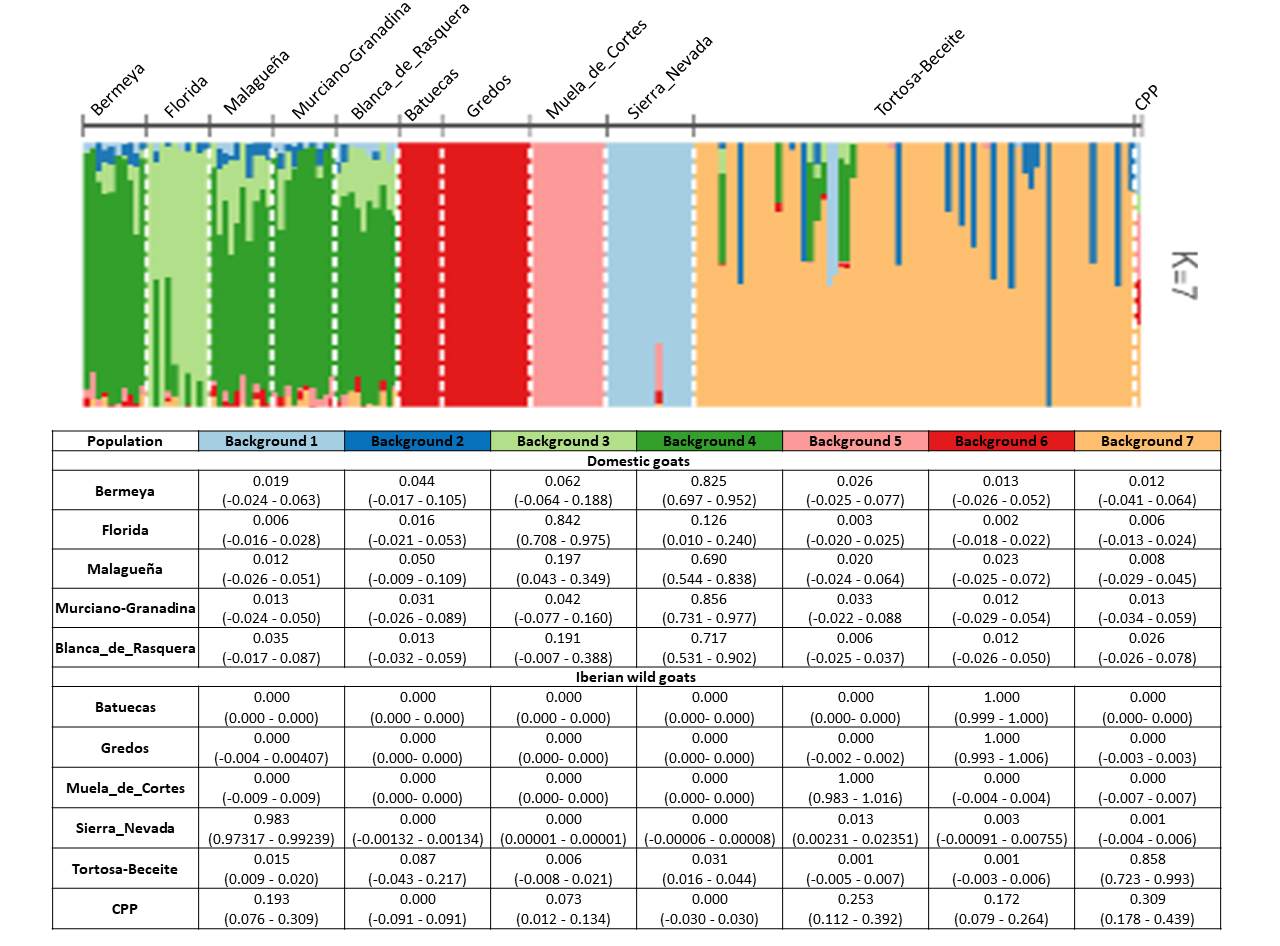

Supplement: Supplementary file 1 — Supplementary Material [file EVA-14-2618-s001.docx]
